# Stem Cell-Like Properties of the Endometrial Side Population: Implication in Endometrial Regeneration

**DOI:** 10.1371/journal.pone.0010387

**Published:** 2010-04-28

**Authors:** Hirotaka Masuda, Yumi Matsuzaki, Emi Hiratsu, Masanori Ono, Takashi Nagashima, Takashi Kajitani, Toru Arase, Hideyuki Oda, Hiroshi Uchida, Hironori Asada, Mamoru Ito, Yasunori Yoshimura, Tetsuo Maruyama, Hideyuki Okano

**Affiliations:** 1 Department of Physiology, Keio University School of Medicine, Tokyo, Japan; 2 Department of Obstetrics and Gynecology, Keio University School of Medicine, Tokyo, Japan; 3 Central Institute for Experimental Animals, Kanagawa, Japan; University of Córdoba, Spain

## Abstract

**Background:**

The human endometrium undergoes cyclical regeneration throughout a woman's reproductive life. Ectopic implantation of endometrial cells through retrograde menstruation gives rise to endometriotic lesions which affect approximately 10% of reproductive-aged women. The high regenerative capacity of the human endometrium at eutopic and ectopic sites suggests the existence of stem/progenitor cells and a unique angiogenic system. The objective of this study was to isolate and characterize putative endometrial stem/progenitor cells and to address how they might be involved in the physiology of endometrium.

**Methodology/Principal Findings:**

We found that approximately 2% of the total cells obtained from human endometrium displayed a side population (SP) phenotype, as determined by flow cytometric analysis of Hoechst-stained cells. The endometrial SP (ESP) cells exhibited preferential expression of several endothelial cell markers compared to endometrial main population (EMP) cells. A medium specific for endothelial cell culture enabled ESP cells to proliferate and differentiate into various types of endometrial cells, including glandular epithelial, stromal and endothelial cells *in vitro*, whereas in the same medium, EMP cells differentiated only into stromal cells. Furthermore, ESP cells, but not EMP cells, reconstituted organized endometrial tissue with well-delineated glandular structures when transplanted under the kidney capsule of severely immunodeficient mice. Notably, ESP cells generated endothelial cells that migrated into the mouse kidney parenchyma and formed mature blood vessels. This potential for *in vivo* angiogenesis and endometrial cell regeneration was more prominent in the ESP fraction than in the EMP fraction, as the latter mainly gave rise to stromal cells *in vivo*.

**Conclusions/Significance:**

These results indicate that putative endometrial stem cells are highly enriched in the ESP cells. These unique characteristics suggest that ESP cells might drive physiological endometrial regeneration and be involved in the pathogenesis of endometriosis.

## Introduction

Human endometrium, which lines the uterine cavity, exhibits unique properties of cyclical regeneration and tissue breakdown under the influence of estrogen and progesterone throughout the course of a woman's reproductive life. Retrograde shedding and ectopic implantation of menstrual endometrial cells and tissue fragments give rise to endometriotic lesions outside of the uterus. Endometrial cells prepared from the human endometrium are also capable of reconstituting functional endometrium in xenograft models of endometriosis [1]. When single cell suspensions of endometrial cells are transplanted under the kidney capsule of severely immunodeficient NOD/SCID/γ_c_^null^ (NOG) mice [1], the reconstructed ectopic endometrial tissues show menstrual cycle-related morphological and functional changes repeatedly in response to treatment with estrogen and progesterone [1]. These unique properties reflect the remarkable capacity of human endometrial cells for regeneration at eutopic and ectopic locations, and suggest the existence of stem/progenitor cells as well as an angiogenic system in the human endometrium. Indeed, it has been postulated that the endometrium contains a pool of multipotent stem cells within the deep *basalis* layer, capable of cyclically producing progenitor cells that further differentiate into each endometrial cell component [2], [3].

Several groups have identified a number of endometrial cell subpopulations as candidate endometrial stem/progenitor cells. These include clonogenic endometrial cells [4], endometrial SP cells which possess a Hoechst 33342 low-fluorescence profile [5], [6], CD146^+^PDGFRβ^+^ stromal cells [7], and CD29^+^CD73^+^CD90^+^ stromal cells [8]. The phenotypic and functional stem cell-like properties, however, have only been characterized *in vitro*. No studies have yet explored the *in vivo* regenerative capacity of these putative endometrial stem/progenitor cells. Candidate tissue-specific stem cells have been identified in several tissues based on the SP phenotype. This characteristic is due to the unique ability of the primitive cells to pump out the DNA binding dye Hoechst 33342 via the ATP-binding cassette transporter G2 (ABCG2) [9]–[12]. Primitive hematopoietic precursors from bone marrow were the first SP cells identified with this technique [13]. We recently demonstrated that SP cells isolated from the human uterine myometrium regenerate human myometrial tissues *in vivo* when xenotransplanted into the uteri of NOG mice [14].

In the present study, we adapted our *in vivo* regeneration assay and SP isolation procedure to characterize the properties of human endometrial SP (ESP) cells. These cells were able to differentiate into endometrium-like tissue and a variety of endometrial cell components when xenotransplanted into NOG mice. This is the first *in vivo* evidence in support of the existence of stem/progenitor cells in the ESP.

## Results

### Isolation of ESP and endometrial main population (EMP) cells from human cycling endometrium

**Figure 1:**
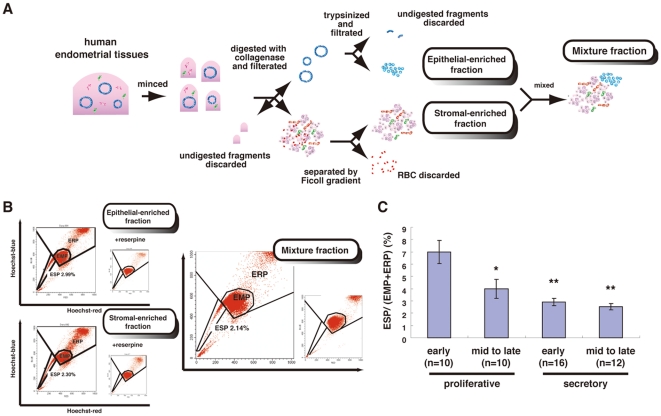
Isolation of ESP and EMP cells. (**A**) Summary of procedures for the preparation of epithelial-enriched and stromal-enriched fractions and the mixture of both fractions from human cycling endometria. (**B**) Flow cytometric distribution of ESP cells, EMP cells, and the endometrial replicative population (ERP) in each of the three fractions stained with Hoechst 33342 indicated in Figure S1. Addition of 50 µM reserpine resulted in the disappearance of the ESP fraction (inset in each panel). (**C**) Proportion of ESP in the whole fraction dissociated from human endometria at different phases of the menstrual cycle. * *P*<0.01 and ** *P*<0.00005, versus early proliferative phase. Each bar indicates the mean ± SEM. n = 48.

We first dissociated human endometria mechanically and enzymatically and purified epithelial-enriched and stromal-enriched fractions as previously described [1]. Procedures for preparing these two fractions are summarized schematically in Figure 1A. Both fractions were then stained with Hoechst dye and subjected to flow cytometric analysis and cell sorting.

We found that each preparation contained a small subset of cells in the SP fraction (Figure 1B). SP cells constituted 2.741±0.443% (mean ± SEM, n = 43) of the viable cells in the epithelial-enriched fraction, whereas SP cells represented 3.091±0.439% (mean ± SEM, n = 43) of the stromal-enriched preparation. The appearance of the SP populations was blocked by 50 µM reserpine (Figure 1B, insets), a general characteristic of SP cells [15]. Since it was unclear which SP fraction contained the endometrial stem/progenitor cells, we mixed the epithelial and stromal SP cells or isolated SP cells from the mixture of the two fractions. We designated the SP and main population (MP) cells derived from both fractions as ESP and EMP, respectively. Endometrial replicative population was designated as ERP (Figure 1B). The ESP and EMP cells were then used for further experiments. The ESP cells represented 2.832±0.326% (mean ± SEM, n = 52) of the total living endometrial cells (Figure 1B).

We next examined whether the proportion of ESP cells varied across the menstrual cycle. As shown in Figure 1C, the proportion of ESP cells to the EMP + ERP fraction was the highest at the early proliferative phase, decreasing gradually until its nadir in the late secretory phase. This may reflect an increase in the number of EMP cells from menstruation towards the late secretory phase.

### *In vivo* reconstitution activity of ESP and EMP cells

**Figure 2:**
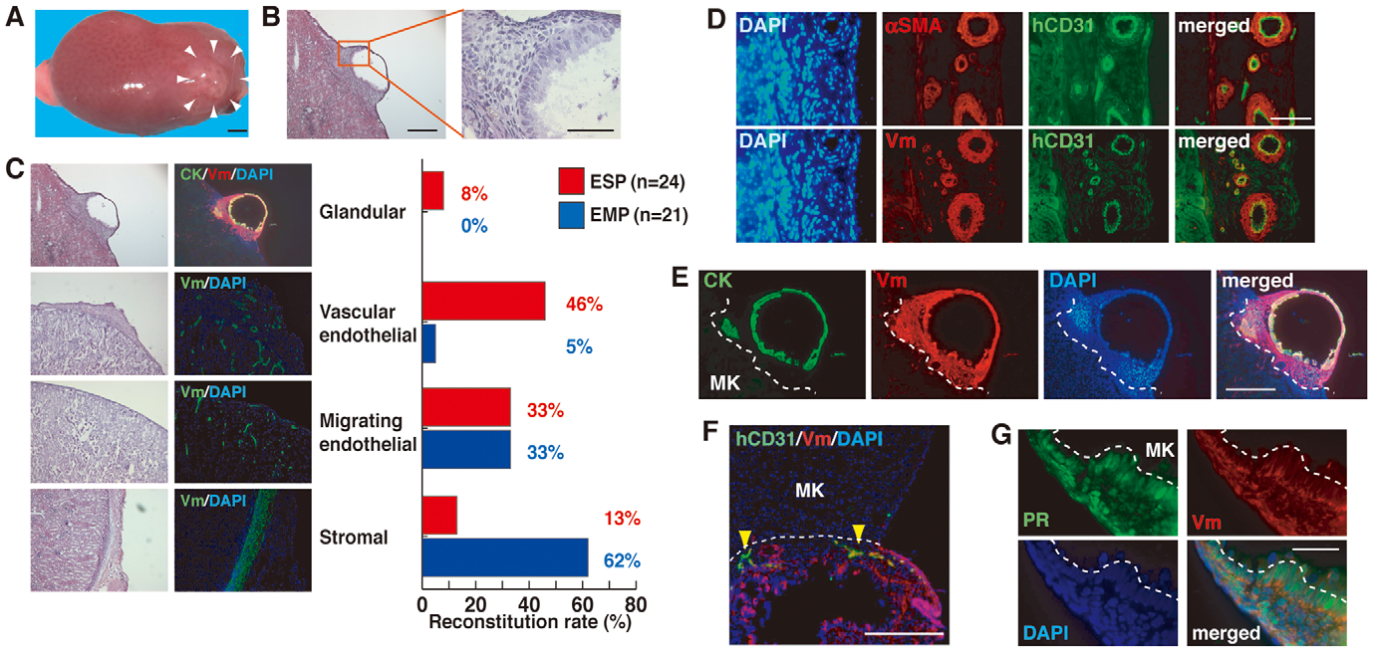
Recreation of the human endometrium and its components from ESP cells in immunodeficient mice. (**A**) The macroscopic appearance of an ESP-initiated lesion (surrounded by white arrowheads) in the kidney of a NOD/SCID/γ_c_^null^ (NOG) mouse treated with E_2_ pellets for 10 weeks. Bar, 1 mm. (**B**) H&E-stained section of the same lesion indicated in (**A**). A small box marks a region shown at a higher magnification in the adjacent panel as indicated. Bars, 500 µm (left) and 100 µm (right). (**C**) H&E-stained and immunofluorescence images of ESP- or EMP-initiated lesions co-stained with DAPI and antibodies against CK and Vm where various endometrial cell components were formed. The right table shows the number and frequency of the ESP- or EMP- initiated mice which predominantly possessed human endometrium with glandular structures, vessel-like structures consisting of endothelial cells, migrating endothelial cells, or stromal cell components. Bars, 500 µm. (**D**) Immunofluorescence images of serial sections of the ESP-initiated lesion co-stained with DAPI and antibodies against αSMA and hCD31. Note that ESP-initiated vessel-like structures positive for hCD31 and Vm were surrounded by muscle-like layers positive for αSMA and Vm. Bars, 100 µm. (**E**) Immunofluorescence images of the same lesion as (**A**) co-stained with DAPI and antibodies against CK and vimentin (Vm). Bars, 500 µm. The borders between the reconstituted tissue and the mouse kidney (MK) are indicated by the dotted lines. (**F**) Immunofluorescence images of the ESP-initiated lesion co-stained with DAPI and antibodies against human CD31 (hCD31) and Vm in NOG mice. Yellow arrowheads indicate hCD31-positive cells. Bars, 200 µm. (**G**) Immunofluorescence images of the ESP-initiated lesion co-stained with DAPI and antibodies against PR and Vm. Bars, 50 µm.

To investigate the stem cell-like regenerative capabilities of ESP cells *in vivo*, we transplanted ESP cells under the kidney capsule of ovariectomized NOG mice, after which they were treated with a 17β-estradiol (E_2_) pellet for eight to ten weeks. As was seen in the endometrial regeneration model [1], ESP cells, but not EMP cells, generated a cystic mass (Figure 2A) with delineated glandular and stromal structures at the site of transplantation (Figure 2B). However, the reconstitution efficiency was low in that the reconstitution of epithelial and stromal endometrial-like tissues was observed in 2 out of 24 xenotransplanted mice (Figure 2C).

No macroscopically identifiable masses were generated in the remaining 22 mice transplanted with ESP cells; however, histological and microscopic analyses of the transplantation sites revealed the existence of three distinctive tissue subtypes within this area. Immunofluorescence studies revealed that the newly formed tissue, but not the mouse kidney, stained with human vimentin (Vm) antibody (clone V9) (Figure 2C). As V9 can recognize only human Vm (hVm), the new tissue was clearly of human origin. These we designated as the “vascular endothelial type”, “migrating endothelial type” and “stromal type”, based on the dominant type of human-originated structures or cell components present in the transplanted site (Figure 2C). In the “vascular endothelial type”, vessel-like structures consisting of hVm^+^ cells were dominant, together with some hVm^+^ cells migrating into the mouse kidney parenchyma (Figure 2C). We confirmed that most of the migratory hVm^+^ cells were positive for CD31 (Figure S1A). Furthermore, ESP-derived CD31^+^ endothelium was surrounded by a layer of ESP-derived smooth muscle cells positive for α-smooth muscle actin (αSMA) (Figure 2D). In the “migrating endothelial type”, migratory cells positive for both hVm and CD31 were dominant in the interspaces of the murine kidney parenchyma (Figure S1A). This type was frequently accompanied by stromal cell layers but not by vessel-like structures. These results collectively suggest that ESP cells have the potential to differentiate into not only small capillary vessels but also mature arteries and veins. The “stromal type” showed layers of fibroblastic hVm^+^ cells, which were also positive for an endometrial stromal cell marker, CD13 (Figure S1B). Notably, the heterogeneity of the regenerated tissue or cell components was more evident in kidneys transplanted with ESP cells than in those transplanted with EMP cells. This indicates that ESP cells may have a broader differentiative capability than cells in the EMP fraction (Figure 2C), as the latter mainly gave rise to stromal cells *in vivo*.

The glandular structure of the well-organized tissue was positive for cytokeratin (CK), an epithelial marker (Figure 2E). The regenerated tissue also contained human CD31-positive endothelial cells (Figure 2F). It is well known that the endometrial progesterone receptor (PR) is upregulated by E_2_ stimulation. In agreement, PR was expressed prominently in the E_2_-exposed endometrial tissue (Figure 2G). These results collectively suggested that ESP cells had the potential to regenerate functional endometrium; however, the efficiency of this process was low (Figure 2F). Indeed, out of the 24 mice xenotransplanted with ESP, only two mice displayed reconstitution of endometrium-like tissue with a glandular structure, which we designated as “glandular type” (Figure 2C).

### Growth and differentiation potentials of ESP and EMP cells

**Figure 3:**
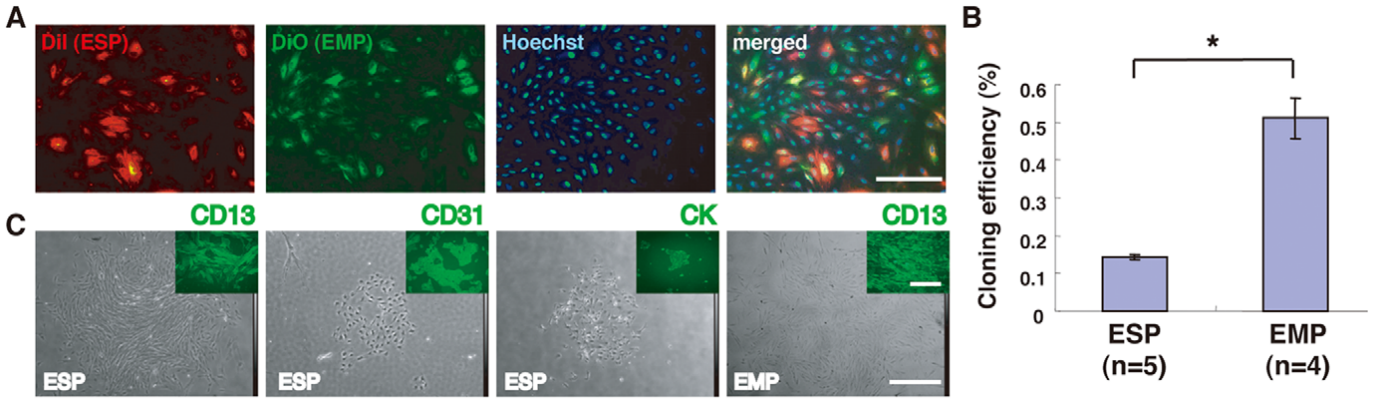
Growth and differentiation potentials of ESP and EMP cells. (**A**) Fluorescence images of DiI-labeled ESP cells co-cultured with DiO-labeled EMP cells following Hoechst DNA dye staining. Bars, 200 µm. (**B**) Cloning efficiencies of ESP cells and EMP cells in EGM-2MV medium. * *P*<0.001. Each bar indicates the mean ± SEM. N ≥4. (**C**) Phase contrast micrographs and fluorescence images (insets) of colonies generated from ESP and EMP cultures. The ESP cells and EMP cells were separately seeded at a clonal density, cultured in EGM-2MV medium for two weeks, and subjected to immunofluorescence studies using antibodies against the indicated markers. CK, cytokeratin. Bars, 500 µm.

To further characterize ESP cells *in vitro*, we attempted to cultivate and expand the cells using conventional media supplemented with various hormones and/or growth factors; however, ESP cells alone never proliferated efficiently. Furthermore, while SP cells derived from human myometrium are able to proliferate preferentially under hypoxic condition [14], ESP cells were not. When cultured together, however, ESP and EMP cells readily proliferated *in vitro* in conventional media (Figure 3A), suggesting that cell-to-cell interactions and/or EMP cell-derived secretory factors may be a prerequisite for the activation of ESP cells.

In vivo migratory and angiogenic potentials of freshly isolated ESP cells (Figure 2C, 2D, and S1) suggest that they may have endothelial cell (EC)-like properties. Therefore, we tested several media customized for EC culture and finally found that ESP cells were able to proliferate in EGM-2MV in the absence of EMP (Figure 3B and 3C).

A clonogenic assay using EGM-2MV medium revealed that the cloning efficiency of ESP cells (0.143±0.008% [mean ± SEM, n = 5]) was significantly less than that of EMP cells (0.511±0.054% [mean ± SEM, n = 4])(Figure 3B). It has been reported that SP cells isolated from the human endometrium, myometrium and other types of tissues are in the G_0_ phase of the cell cycle [6], [14], [16], [17]. Given the quiescence of ESP cells [6], [14], [16], [17], it is conceivable that our clonogenic assay using short-term (14 days) culture may detect the clonogenic activities of committed progenitors (perhaps stromal progenitors) in EMP cells, but not those of the endometrial stem cells and/or the most primitive progenitors in ESP cells. Elucidation of the precise mechanism(s) awaits further studies.

Immunofluorescence studies revealed three distinct types of colonies in the ESP cultures. The most frequent type was comprised of fibroblastoid stromal cells positive for CD13, an endometrial stromal cell marker (Figure 3C). The second most frequent type displayed a cobblestone appearance and was comprised of CD31-positive cells (Figure 3C), which indicated similarities between these cells and late outgrowing endothelial progenitor cells (EPCs) [18]. The least frequent type of colony formed small nests that were comprised of epithelial cells positive for CK, an endometrial epithelial marker (Figure 3C). In contrast to ESP cultures, most of the colonies in the EMP cultures were comprised of CD13-positive fibroblastic stromal cells (Figure 3C). Therefore, ESP cells and not EMP cells possessed the stem/progenitor cell characteristic of generating many lineages of cell types within the tissue in which they are located.

### Subpopulations of ESP cells and their morphology, surface markers, and localization in the human endometrium

**Figure 4:**
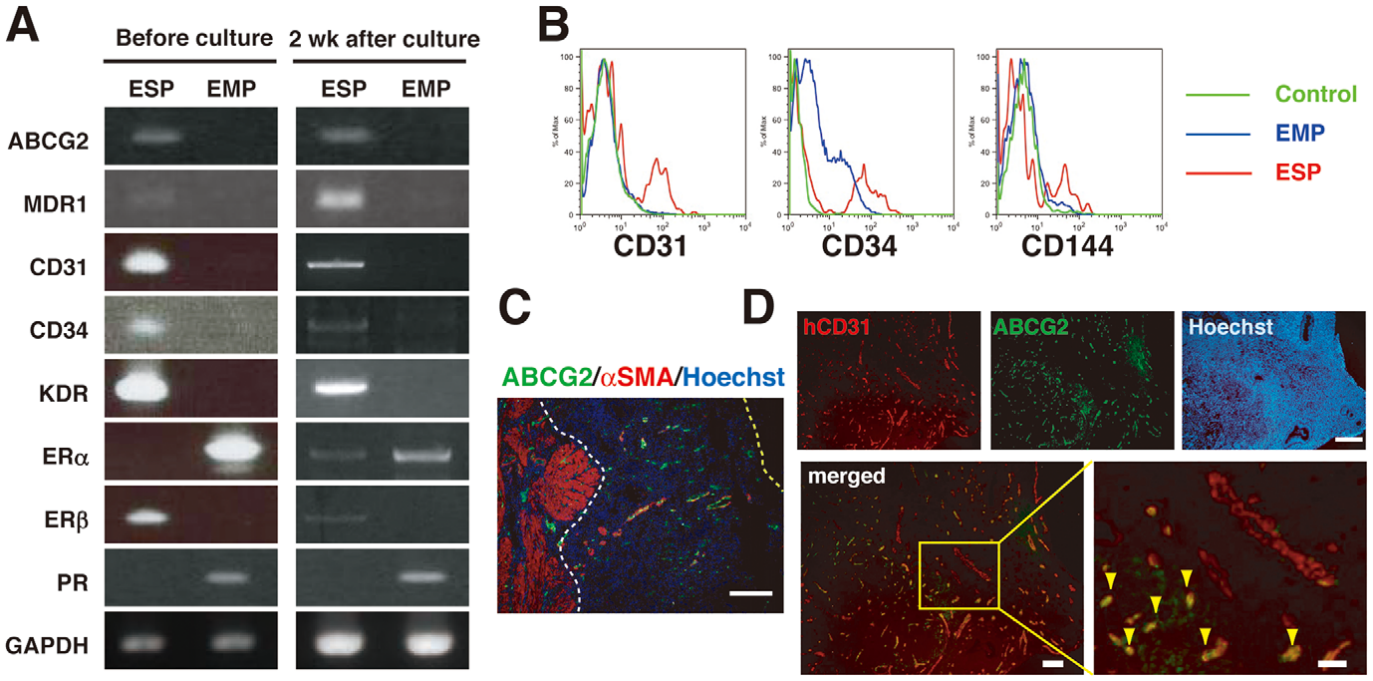
Subpopulations of ESP cells: surface markers, and localization in human endometrium. (**A**) RT-PCR analyses of various transcripts in ESP and EMP cells before and after two weeks of culture. MDR1, multidrug resistance 1; KDR, kinase insert domain-containing receptor. (**B**) Flow cytometric analysis of ESP and EMP cells immunostained with isotypic control antibodies or antibodies against endothelial cell surface markers (CD31, CD34, and CD144). (**C**) An immunofluorescence merged image of human endometrium co-stained with Hoechst and antibodies against ABCG2 and αSMA. White and yellow dotted lines indicate the endometrium-myometrium junction and the luminal surface of the uterine epithelium, respectively. Bars, 200 µm. (**D**) Immunofluorescence images of human endometrium co-stained with Hoechst and antibodies against hCD31 and ABCG2. A small box marks a region shown at a higher magnification in the adjacent panel as indicated. Yellow arrowheads indicate endothelial cells doubly positive for hCD31 and ABCG2. Bars, 200 µm (upper), 100 µm (lower left) and 50 µm (lower right).

To further characterize ESP cells, gene expression analysis of ESP and EMP cells was performed using RT-PCR. ESP cells preferentially expressed mRNA of universally SP-associated markers, ABCG2 and multidrug resistance 1 [15], [19]. ESP cells also expressed CD31, CD34 and KDR (Figure 4A), consistent with the EC-like properties of ESP cells. Interestingly, estrogen receptor (ER) β was abundant in ESP, while neither ERα nor PR was detected. ERβ is also preferentially expressed in the vascular endothelial cells of the human endometrium [20]. The expression of these markers in ESP cells did not change after two weeks of culture (Figure 4A), except for a slight increase and decrease in ERα and ERβ mRNA expression, respectively. This indicates that EGM-2MV medium has the potential to maintain the undifferentiated status of ESP.

Flow cytometric analysis of ESP cells from whole endometrium stained with CD31, CD34, and CD144 antibodies, revealing that ESP cells preferentially expressed these EC-associated markers (Figure 4B). Thus, the expression pattern of the surface markers, the cobblestone-like cell morphology in EGM-2MV medium and the *in vivo* capacity to preferentially give rise to ECs (Figure 3C, 3D and 3F) are all properties of ESP cells that are shared with EC or EPC. In terms of hematopoietic or mesenchymal stem cell markers, our flow cytometric data showed that some SP cells express CD34, CD90, CD105 or CD146, but do not CD133 (Figure S2), and ESP is not a homogeneous population.

Since ESP cells, like other SP cells, expressed the SP-specific marker ABCG2 [15], we performed immunofluorescence staining of human cycling endometrium using ABCG2 antibody to address the tissue localization of ESP cells. The putative endometrial stem/progenitor cells are believed to reside in the *basalis* layer of the human endometrium. Given that the ESP contains candidate endometrial stem/progenitor cells, we anticipated ABCG2^+^ cells might be predominantly located in the *basalis* layer. Unexpectedly, however, ABCG2^+^ cells were evenly distributed across both the *functionalis* and *basalis* layers of the endometrium (Figure 4C).

Taking into account the expression of CD31 in the ESP, we performed double immunofluorescence staining using CD31 and ABCG2 antibodies to examine the localization of CD31^+^ ESP cells in human cycling endometrium. We found that CD31^+^ABCG2^+^ cells, presumably containing CD31^+^ ESP cells, were preferentially located in small capillaries, rather than large vessels (Figure 4D). Again, CD31^+^ABCG2^+^ cells were present throughout the *functionalis* and *basalis* layers.

## Discussion

Human and primate endometrium regenerates from the lower *basalis* layer, a germinal compartment that persists after menstruation to give rise to the new upper *functionalis* layer [2], [21]–[23]. The surface epithelium develops primarily through the proliferation of epithelial cells from the tips of the gland stumps [24], [25]. The findings presented here strongly support the idea that the basalis of the endometrium harbors stem/progenitor cells responsible for endometrial regeneration during menses as well as after parturition in both women and menstruating non-human primates [23]. It remains possible, however, that endometrial stem/progenitor cells also exist in the *functionalis* of the endometrium. Indeed, the ABCG2^+^ population, which presumably includes ESP cells having endometrial stem cell-like properties (present study and [5]), is localized exclusively in the endothelium of both the functional and basal layers of the human endometrium (Figure 3 and [6]). A relatively small number of dispersed human endometrial cells (mainly derived from the *functionalis* layer of the endometrium) containing ESP cells can generate functional endometrial tissue comprising glands, stroma, immune cells and vascular components when they are transplanted under the kidney capsule of severely immunodeficient mice [1]. Mesenchymal stem-like cells expressing both CD146 and PDGF-Rβ are located perivascularly in the *functionalis* and *basalis* layers of the human endometrium [7]. Very recently, Garry *et al.* demonstrated that endometrial surface epithelial regeneration takes place as a consequence of cellular differentiation from stromal cells and not by direct extension from the residual basal epithelial glands [26]. These findings collectively suggest that ESP cells present in the vascular endothelium are one of the most likely candidates for endometrial stem/progenitor cells that may reside not only in the *basalis* but also in the *functionalis* endometrium.

The percentage of SP cells derived from cultured epithelial-enriched fraction was significantly greater during menstruation than at any other cycle stage [5], whereas the rate of SP cells freshly isolated from endometrial samples was significantly greater in the proliferative phase than in the secretory phase [6]. In this study we showed that the proportion of ESP cells to the EMP + ERP fraction freshly isolated from human endometria was the highest at the early proliferative phase among all the phases of the menstrual cycle, which is in agreement with previous report [6], and it decreased gradually until its nadir in the late secretory phase. Given the stem cell-like properties of ESP cells as presented here and reported elsewhere [5], [6], it is tempting to speculate that ESP cells may generate committed progenitor cells through asymmetrical cell division, which, in turn, may lose the SP phenotype, further behave as transient amplifying cells, and eventually propagate through symmetrical cell division. In this context, the gross number of ESP cells may be almost unchanged, but the number of non-SP cells (EMP + ERP cells) present particularly in the *functionalis* endometrium may increase, presumably resulting in the decline in the proportion of ESP cells from menstruation towards the late secretory phase.

There are several explanations for the low efficiency of reconstitution. First, the local environment at the xenotransplantation site may lack necessary factors for the regeneration of the entire endometrium. Second, ESP cells may require a specific “niche” provided by other endometrial cell components to reconstitute the entire endometrium *in vivo* as well as *in vitro* culture. Successful proliferation of ESP cells in the presence of EMP cells in conventional media (Figure 3A) suggests that EMP cells may provide a “niche” appropriate for activation of ESP cells. Moreover, successful proliferation of ESP cells alone in EGM-2MV medium prompts us to postulate that EMP cells alone and/or cell-to-cell interaction between ESP and EMP cells may produce bioactive substances such as EGF, VEGF, bFGF, and/or insulin-like growth factor-I that EGM-2MV medium contains. Microenvironments under the kidney capsule of NOG mice may also provide some but not sufficient “niche”, which may be at least in part attributed to low efficiency of reconstitution of organized endometrial tissue from ESP cells. We have previously demonstrated that the single cell suspension without any cell selection which consisted of ESP cells and non-SP cells could reconstitute well-organized endometrial tissue at almost 100% frequency [1], which is much greater than the rate of ESP cells alone (8%). These findings suggest that EMP cells are required as a “niche” provider for *in vivo* tissue reconstitution but EMP cells alone may not be able to generate the organized endometrial tissues *in vivo*. Third, ESP cells might not contain a sufficient number of stem/progenitor cells for generating glandular cells. In this study, we collected endometrial tissues by scraping strongly the uterine cavity with the back edge of a scalpel. A portion of the endometrium, particularly the deep *basalis* layer, however, penetrates into the myometrium where it will not be collected by scraping (Figure S3). Thus, it is possible that some of the ESP cells present in the deep *basalis* layer may not be included in the starting endometrial materials in this study.

In this study, we have shown that at least some ESP cells were localized to the endometrial endothelial wall, predominantly expressed several EC markers, preferentially proliferated and differentiated *in vitro* in an EC-specific medium, and displayed high migratory and angiogenic potential. These results suggest that ESP cells have endometrial stem cell-like properties as well as EC or EPC-like characteristics. EPCs are believed to be derived from the bone marrow and to home to sites of neovascularization and neoendothelialization where they differentiate into ECs [27], [28]. This raises the possibility that ESP may have originated from bone marrow stromal cells. Indeed, bone marrow-derived EPCs contribute to the formation of new blood vessels in human and mouse endometrium [29], [30]. Furthermore, bone-marrow derived cells give rise to uterine epithelial cells in humans [31] and mice [32], [33], although the identity of these cells remains unclear. Based on the present results, we speculate that ESP represents one such candidate population.

**Figure 5:**
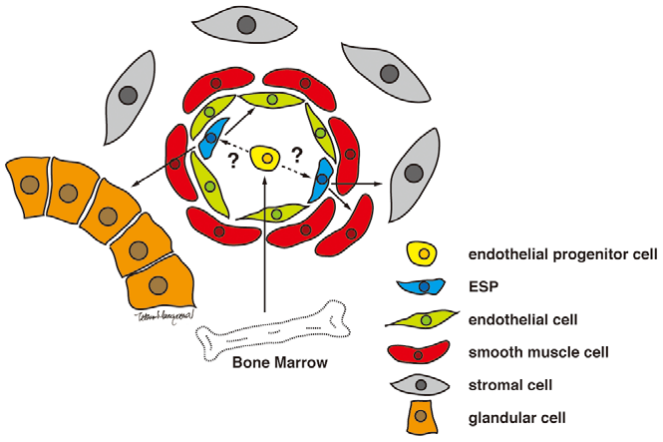
Proposed model for ESP-driven endometrial regeneration and the establishment and progression of endometriosis.

In view of these findings, we here propose a single model for ESP-driven endometrial regeneration and establishment of endometriosis (Figure 5). In this model, ESP cells, perhaps ultimately derived from the bone marrow, mainly reside in vascular endothelial walls and/or perivascular regions. Importantly, these ESP cells are present not only in the *basalis* but also in the *functionalis* endometrium. These cells, therefore, might be contained within the sloughed endometrium shed at menstruation. They might then implant onto the surface of ectopic sites such as the peritoneum through retrograde menstruation. Furthermore, some of these *functionalis* layer-derived ESP cells might remain in the uterine cavity after menstruation and implant again (reimplant) onto the deconstructed eutopic endometrium. In both eutopic and ectopic implantation, endothelial ESPs might give rise to various endometrial cell components in the process of ESP-driven angiogenesis. Our eutopic reimplantation hypothesis does not contradict the current paradigm but rather provides an additional mechanism for endometrial regeneration. We describe previously that a certain type of cells in endometrium could migrate, invade, form chimeric vasculature in the host kidney of NOG mouse and establish the functional circulatory system[1]. In terms of the ability to invade into kidney parenchyma, these cells could be SP cells. From this point, their ability may be crucial for establishment and development endometriosis, because the angiogenesis is absolutely required for maintenance of endometriotic lesion. In support of this idea, a stem cell theory of the pathogenesis of endometriosis has been recently emerged [34], postulating that endometrial stem/progenitor cells may function in the development of endometriosis. Our proposed new model suggests that only a small population of endometrial cells, specifically ESP cells, has the potential to generate endometriotic lesions through their unique migratory and angiogenic activities and provides a proving ground of anti-angiogenic therapy recently proposed as a potential treatment for endometriosis [35]–[37].

We have shown that ESP cells, but not EMP cells, express ERβ but do not express ERα or PR. A similar expression pattern is observed in endometrial vascular ECs which express ERβ, but neither ERα nor PR [20]. Those findings further substantiate our present results that ESP cells have EC-like properties and reside in the endometrial endothelium. Finally, the expression level of ERβ is strikingly higher than those of ERα and PR in endometriotic lesions [38], implicating ERβ-positive ESP cells in the pathogenesis of endometriosis.

In summary, we have demonstrated that undifferentiated ESP cells are present in human cycling endometrium. Purified ESP cells, but not EMP cells, contain putative endometrial stem/progenitor cells with potentials for differentiation into multiple types of endometrial cells *in vitro* and *in vivo*. In our hands, ESP cells were not able to proliferate from a single cell *in vitro*. Without single cell analyses, it remains uncertain whether individual ESP cells posses multi-lineage differentiation potential at the clonal level or, instead, the cells consists of a mixed population of progenitors or stem cells. The study of the ESP, however, will improve the understanding of endometrial physiology and provide insight into the pathogenesis and treatment of endometrium-derived diseases such as endometriosis.

## Materials and Methods

Detailed protocols can be found in Methods S1.

### Tissue collection

Endometrial specimens (n = 78) were collected from women with normal menstrual cycles undergoing total abdominal hysterectomy for benign gynecological diseases, or cervical carcinoma *in situ*. Written informed consent was obtained from each patient and the use of these human specimens was approved by the Keio University Ethics Committee.

### Isolation and flow cytometric analysis of ESP and EMP cells

Endometrial specimens were separated and dissociated into endometrial stromal and glandular epithelial single cell fractions as described previously [1]. Stromal-enriched and epithelial-enriched fractions were washed in calcium- and magnesium-free HBSS supplemented with 2% FBS, 10 mM HEPES buffer, and 1% penicillin-streptomycin (HBSS^+^) and suspended at 2×10^6^ cells/mL in HBSS^+^ and stained with 5.0 µg/mL Hoechst33342 (Sigma Chemical, St. Louis, MO) for 90 min at 37°C, as described previously [12], [13]. Fluorescein isothiocyanate, phycoerythrin or allophycocyanin-conjugated antibodies for flow cytometry and propidium iodide were simultaneously added to Hoechst-stained cells suspended in HBSS^+^. Cells were incubated on ice for 30 minutes, pelleted, and washed with HBSS^+^. The antibodies we used are listed in Table S1. Flow cytometric analysis and cell sorting were performed as described in *SI Methods*. After collecting 1×10^5^ events, the SP population was defined as previously reported [13].

### Co-culture of SP and MP cells

To track the fate of SP cells in co-culture with MP cells, the SP and MP cells were labeled with two different fluorescent dyes-Vybrant DiO (green fluorochrome) and Vybrant DiI (red fluorochrome) (Molecular Probes, Eugene, OR). These reagents allow two-color labeling of cell populations for identification after mixing and co-culture. DiI-labeled SP cells and DiO-labeled MP cells were mixed at a ratio of 1∶1 and co-cultured in DMEM containing 1% antibiotic-antimycotic, and 10% FBS.

### Cell culture and determination of cloning efficiency

SP and MP cells were separately seeded at a clonal density of 400 cells/cm^2^ in 35 mm dishes and cultured in EGM-2MV medium (Cambrex, Walkersville, MD). After 14 days of culture, clusters of cells were considered colonies when they were visible macroscopically and had more than 50 cells. Colonies were counted and the cloning efficiency (CE) was determined from the formula: CE (%)  =  (number of colonies/number of cells seeded) ×100.

### RT-PCR

Total RNA was extracted from cell cultures and subjected to RT-PCR. First-strand cDNA was synthesized and amplified using specific PCR primers (Table S2). The PCR products were separated by electrophoresis on agarose gels and visualized by ethidium bromide staining with UV light illumination.

### Xenotransplantation and hormonal treatment

The same numbers of SP and MP cells (10^4^ to 10^5^ cells), freshly isolated from human endometria, were immediately transplanted under the kidney capsules of NOG mice as described previously [1].

### Histology and immunohistochemistry

H&E-staining and immunofluorescence analyses were performed on culture dishes or cryosections derived from kidneys transplanted with ESP or EMP, that were air-dried, washed, and fixed. After permeabilization and blocking, tissue sections were incubated with the pretitrated primary antibodies listed in Tables S1 and S3. For indirect fluorescence staining, the first antibodies were visualized by incubation with secondary antibodies conjugated with Alexa Fluor 488 (green) or 568 (red) (Molecular Probes). We classified the histological feature of the reconstituted tissue and its adjacent transplantation site into four subtypes (i.e., glandular, vascular endothelial, migrating endothelial, and stromal), based on their dominant type (Figure 2C). The reconstitution rate of ESP and EMP cells was determined by the following formula: Reconstitution rate (%)  =  (number of a corresponding subtype/number of transplanted kidneys) ×100. Images were collected as described in *SI Methods*.

### Statistics

Results are expressed as means ± SEM. Comparisons among the SP rates for each of the four phases were made with a Tukey test and the others were done using the unpaired Student's t test. P values less than 0.05 were considered statistically significant.

## Supporting Information

Figure S1 Expression of endothelial and stromal cell markers in human-derived cells present around the ESP-initiated lesion. Immunofluorescence images of the ESP-initiated lesion in NOG mouse kidney co-stained with DAPI and antibodies against Vm and hCD31 (A) or antibodies against Vm and CD13 (B). Bars, 100 µm.
(4.16 MB TIF)

Figure S2 Expression of hematopoietic stem cell marker and mesenchymal stem cell marker on ESP cells. Flow cytometric analysis of ESP cells stained with antibodies against hematopoietic stem cell markers (CD34 and CD133) and mesenchymal stem cell markers (CD90, CD105 and CD146).
(3.15 MB TIF)

Figure S3 Remaining basalis layer of endometrium after endometrial tissue has been scraped off. Histological and immunofluorescence images of the uterine interface between the endometrium and myometrium stained with H&E or antibodies against CK and αSMA. Bars, 500 µm. Black and yellow arrowheads indicate endometrial glands present adjacent to or inside the myometrium.
(0.96 MB TIF)

Table S1 List of antibodies used for flow cytometric analysis.
(0.03 MB DOC)

Table S2 Sequences of the primers used for detection of various genes.
(0.04 MB DOC)

Table S3 List of antibodies used for immunofluorescence staining.
(0.04 MB DOC)

Methods S1 (0.07 MB DOC)
