# Supplementary figures and images for: Stem Cell-Like Properties of the Endometrial Side Population: Implication in Endometrial Regeneration
Source: PLoS One. 2010 Apr 28;5(4):e10387. doi: 10.1371/journal.pone.0010387 (PMC2860997; doi:10.1371/journal.pone.0010387)

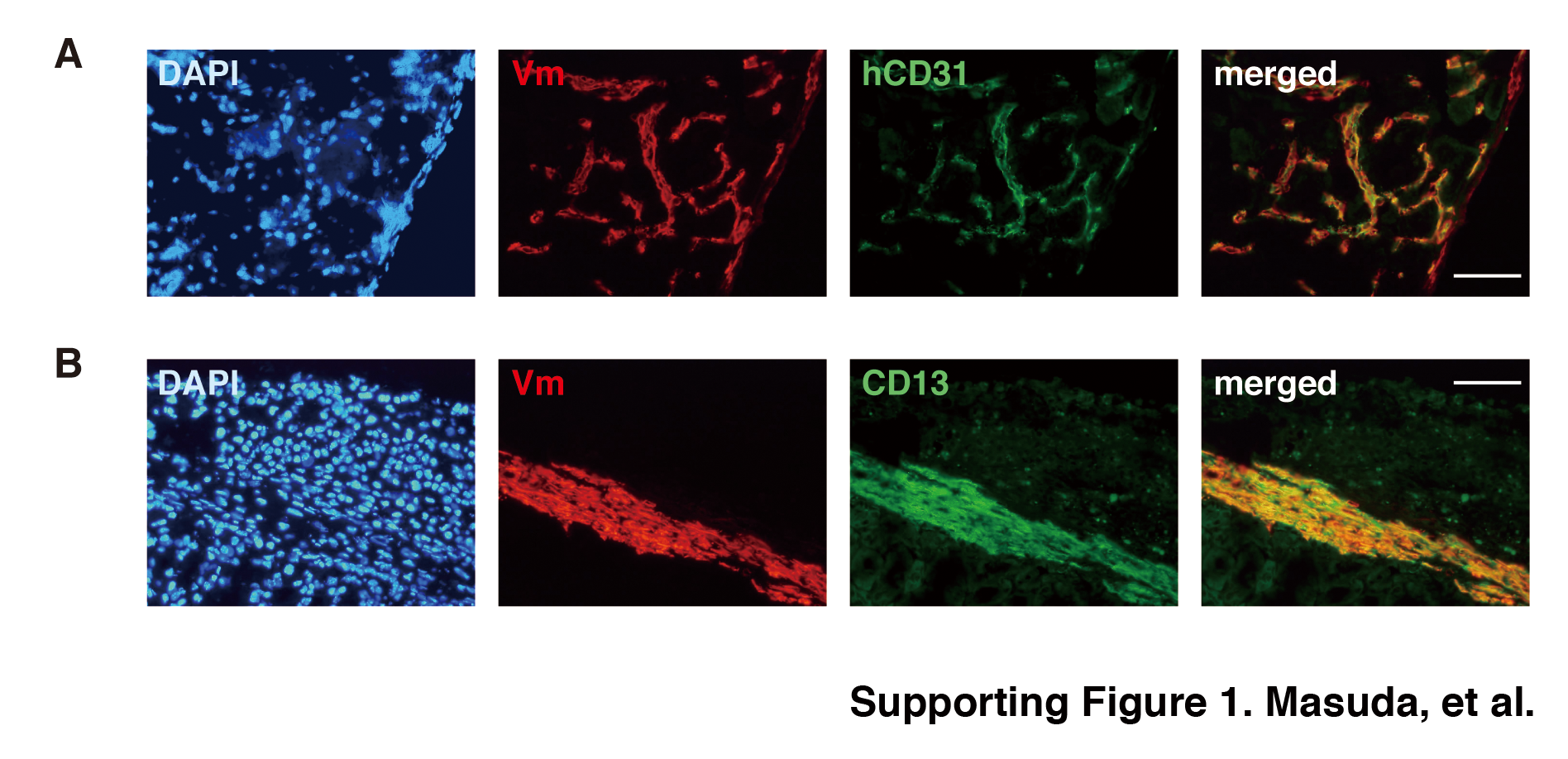

Supplement: Figure S1 — Expression of endothelial and stromal cell markers in human-derived cells present around the ESP-initiated lesion. Immunofluorescence images of the ESP-initiated lesion in NOG mouse kidney co-stained with DAPI and antibodies against Vm and hCD31 (A) or antibodies against Vm and CD13 (B). Bars, 100 µm. (4.16 MB TIF) [file pone.0010387.s001.tif]

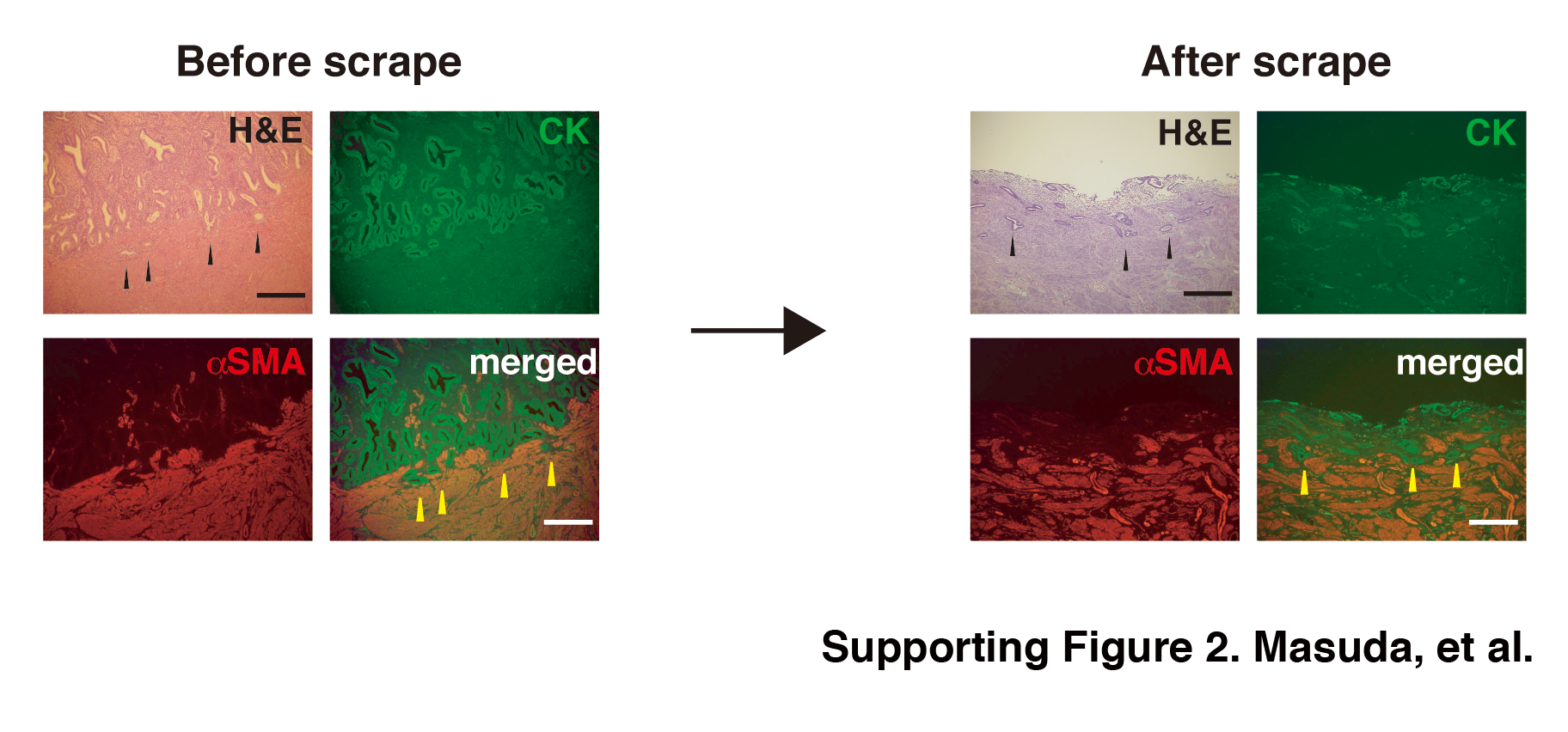

Supplement: Figure S2 — Expression of hematopoietic stem cell marker and mesenchymal stem cell marker on ESP cells. Flow cytometric analysis of ESP cells stained with antibodies against hematopoietic stem cell markers (CD34 and CD133) and mesenchymal stem cell markers (CD90, CD105 and CD146). (3.15 MB TIF) [file pone.0010387.s002.tif]

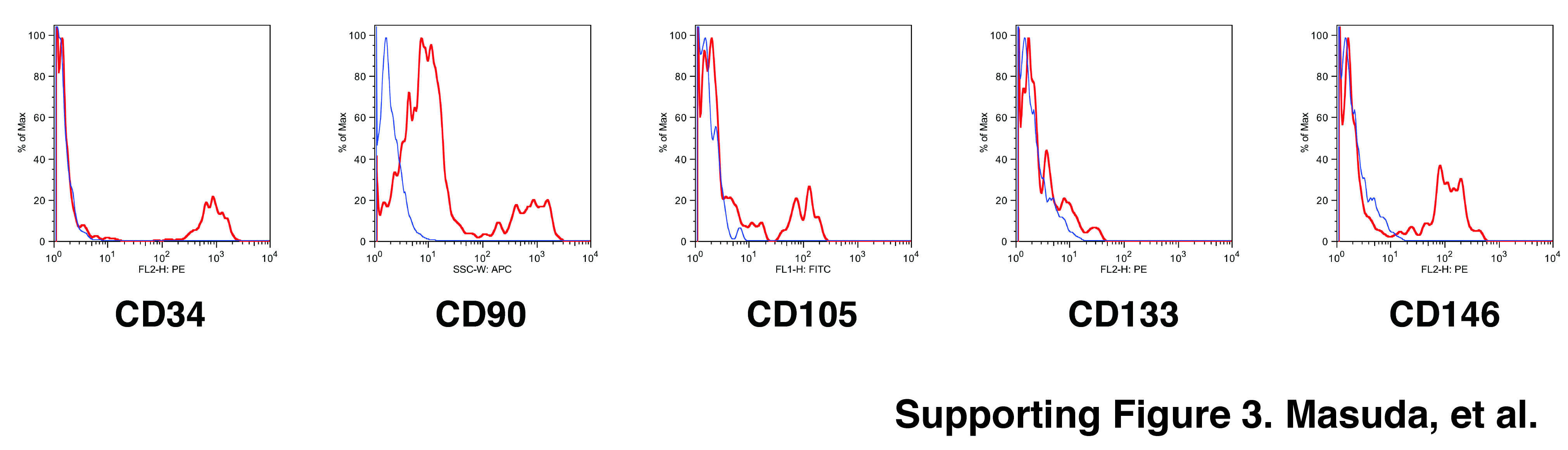

Supplement: Figure S3 — Remaining basalis layer of endometrium after endometrial tissue has been scraped off. Histological and immunofluorescence images of the uterine interface between the endometrium and myometrium stained with H&E or antibodies against CK and αSMA. Bars, 500 µm. Black and yellow arrowheads indicate endometrial glands present adjacent to or inside the myometrium. (0.96 MB TIF) [file pone.0010387.s003.tif]
