# Supplementary material for: Stem Cell-Like Properties of the Endometrial Side Population: Implication in Endometrial Regeneration
Source: PLoS One. 2010 Apr 28;5(4):e10387. doi: 10.1371/journal.pone.0010387 (PMC2860997; doi:10.1371/journal.pone.0010387)
Supplement: Table S1 — List of antibodies used for flow cytometric analysis. (0.03 MB DOC) [file pone.0010387.s004.doc]

**Table S1. List of antibodies used for flow cytometric analysis.**

| **Antigen** | **Clone** | **Supplier** |
| --- | --- | --- |
| CD31 | WM59 | BD PharMingen |
| CD34 | 581/CD34 | BD PharMingen |
| CD144 | 16B1 | e-Bioscience |
| ABCG2 | 5D3 | e-Bioscience |
