# Supplementary material for: Stem Cell-Like Properties of the Endometrial Side Population: Implication in Endometrial Regeneration
Source: PLoS One. 2010 Apr 28;5(4):e10387. doi: 10.1371/journal.pone.0010387 (PMC2860997; doi:10.1371/journal.pone.0010387)
Supplement: Table S2 — Sequences of the primers used for detection of various genes. (0.04 MB DOC) [file pone.0010387.s005.doc]

**Table S2. Sequences of the primers used for detection of various genes.**

| **Gene** | **Sequence (5' to 3')** |
| --- | --- |
| GAPDH (F) | CGGAGTCAACGGATTTGGTCGTAT |
| GAPDH (R) | AGCCTTCTCCATGGTGGTGAAGAC |
| ABCG2 (F) | CCAGTTCCATGGCACTGGCCATA |
| ABCG2 (F) | CAAGGCCACGTGATTCTTCCACA |
| MDR1 (F) | GCAAAGCTGGAGAGATCCTCACCA |
| MDR1 (R) | CAACATTTTCATTTCAACAACTCCTGC |
| CD31 (F) | CAAAGACAACCCCACTGAAG |
| CD31(R) | CACTCCGATGATAACCACTG |
| CD34 (F) | AGGTATGCTCCCTGCTCCTGGCCC |
| CD34 (R) | AAGAACAGCCTCTGAGGTGTGTGC |
| ERα (F) | ACATGAGAGCTGCCAACCTT |
| ERα (R) | TCCAGAGACTTCAGGGTGCT |
| ERβ (F) | AAGAAGATTCCCGGCTTTGT |
| ERβ (R) | CTTGTTACTCGCATGCCTGA |
| PR (F) | GATTCAGAAGCCAGCCAGAG |
| PR (R) | TGCCACATGGTAAGGCATAA |
| (F), Forward primer; (R), reverse primer | |
