# Supplementary material for: Stem Cell-Like Properties of the Endometrial Side Population: Implication in Endometrial Regeneration
Source: PLoS One. 2010 Apr 28;5(4):e10387. doi: 10.1371/journal.pone.0010387 (PMC2860997; doi:10.1371/journal.pone.0010387)
Supplement: Table S3 — List of antibodies used for immunofluorescence staining. (0.04 MB DOC) [file pone.0010387.s006.doc]

**Table S3. List of antibodies used for immunofluorescence staining.**

| **Antigen** | **Clone** | **Isotype** | **Supplier** |
| --- | --- | --- | --- |
| cytokeratin | MNF116 ^B^ | mouse IgG | DakoCytomation (Glostrup, Denmark) |
| vimentin | V9 ^A^ | Cy3-conjugated mouse IgG | SIGMA Chemical (St. Louis, MO) |
| vimentin | V9 ^A^ | mouse IgG | DakoCytomation |
| human CD9 | P1/33/2 | mouse IgG | DakoCytomation |
| human CD13 | WM-47 | mouse IgG | DakoCytomation |
| human CD31 | JC70A ^A^ | mouse IgG | DakoCytomation |
| progesterone receptor (PR) | PgR636 | mouse IgG | DakoCytomation |
| α-smooth muscle actin | 1A4 ^B^ | Cy3-conjugated mouse IgG | SIGMA Chemical |
| α-smooth muscle actin | 1A4 ^B^ | mouse IgG | DakoCytomation |
| ABCG2 | BXP-34 | mouse IgG | SIGMA Chemical |
| A: antibodies react with human antigens only. | | |  |
| B: antibodies react with both human and murine antigens. | | |  |
