# Supplementary material for: Stem Cell-Like Properties of the Endometrial Side Population: Implication in Endometrial Regeneration
Source: PLoS One. 2010 Apr 28;5(4):e10387. doi: 10.1371/journal.pone.0010387 (PMC2860997; doi:10.1371/journal.pone.0010387)
Supplement: Methods S1 — (0.07 MB DOC) [file pone.0010387.s007.doc]

## **SI Methods**

## **Tissue collection**

Endometrial specimens (n = 78) were collected from women with normal menstrual cycles undergoing total abdominal hysterectomy for benign gynecological diseases, or cervical carcinoma *in situ*. Written informed consent was obtained from each patient and the use of these human specimens was approved by the Keio University Ethics Committee. There were no abnormalities or malignancies in these specimens as diagnosed by histological examination. The stage of the menstrual cycle was determined based on the date of the last menstrual cycle and the endometrial histology using the established dating criteria of Noyes *et al*. [1].

## ***Preparation of stromal-enriched and epithelial-enriched fractions from human endometrium***

Endometrial specimens were separated and dissociated into endometrial stromal and glandular epithelial single cell fractions as described previously [2]. Stromal cells prepared in a 50 mL tube were pelleted by centrifugation for seven min at 440 × g and resuspended in 4 mL of calcium- and magnesium-free HBSS (GIBCO, Carlsbad, CA) supplemented with 2% FBS (BioWest, Miami, FL), 10 mM HEPES buffer solution (GIBCO) and 1% penicillin-streptomycin (GIBCO) (HBSS^+^). Red blood cells were removed by centrifugation over 3 mL Ficoll-Paque PLUS (Amersham Biosciences, Piscataway, NJ) in a 15 mL tube. The media/Ficoll interface, mainly containing stromal cells, was carefully aspirated, washed, and then subjected to Hoechst staining.

Glandular epithelial fragments were digested with 0.05% **trypsin**-EDTA solution (Sigma-Aldrich, St. Louis, MO) and 0.05% DNAase I by pipetting for 5-10 min to dissociate the **gland**s into single **cell**s. Subsequently, the dispersed glandular cells were washed with DMEM (Sigma-Aldrich) containing 1% antibiotic-antimycotic solution (GIBCO), and 10% FBS (DMEM^+^) and filtered through a 40 μm cell strainer for removal of undigested, adherent glandular tissues to yield a single-cell suspension containing the epithelial-enriched fractions for Hoechst staining.

## ***Isolation and flow cytometric analysis of SP and MP cells***

Stromal-enriched and epithelial-enriched fractions were washed in HBSS^+^ and suspended at 2 × 10^6^ cells/mL in HBSS^+^ and stained with 5.0 μg/mL Hoechst33342 (Sigma Chemical, St. Louis, MO) for 90 min at 37°C, as described previously [3, 4]. Fluorescein isothiocyanate, phycoerythrin or allophycocyanin-conjugated antibodies for flow cytometry and propidium iodide were simultaneously added to Hoechst-stained cells suspended in HBSS^+^. Cells were incubated on ice for 30 minutes, pelleted, and washed with HBSS^+^. The antibodies are listed in Supplemental Table 1. Flow cytometric analysis and cell sorting were performed on a triple laser MoFlo (Cytomation, Fort Collins, CO) with Summit software (Cytomation) or a FACSVantage SE flow cytometer (BD Biosciences, San Jose, CA) with CELLQuest (BD Biosciences) software. Hoechst 33342 was excited at 350 nm, and the fluorescence emission was detected using a 405/BP (band pass) 30 and 570/BP20 optical filters for Hoechst blue and Hoechst red, respectively, and a 550 nm long-pass dichroic mirror (Omega Optical Inc., Brattleboro, VT) to separate the emission wavelengths. Both Hoechst blue and red fluorescence intensities are shown on a linear scale. Propidium iodide fluorescence was measured through 630/BP30 after excitation at 488 nm with an argon laser, and a live cell gate was defined to exclude propidium iodide -positive cells. After collecting 1 × 10^5^ events, the SP population was defined as previously reported [3].

## ***Mice***

NOG mice were established at the Central Institute of Experimental Animals (Kawasaki, Japan) by backcrossing C57BL/6J-γ_c_^null^ mice and NOD/Shi-scid mice. NOG mice accept xenografts with a high success rate due to multiple immunological dysfunctions, including inability to produce cytokines and functional incompetence of T, B, and natural killer cells [5, 6].

## ***Cell culture and determination of cloning efficiency***

SP cells were seeded at 10,000 cells/cm^2^ in dishes (BD labware, Franklin Lakes, NJ) containing one of the following: (1) DMEM^+^, or (2) HBSS^+^, or (3) alpha minimum essential medium (alpha-MEM; Sigma-Aldrich) containing 1% antibiotic-antimycotic solution and 20% FBS (alpha-MEM+), or (4) mesenchymal stem cell basal medium (MSCBM; Cambrex). We tested the following culture conditions. SP cells were cultured in DMEM^+^, or HBSS^+^ or alpha-MEM^+^ in the presence of one or two of the following hormones or growth factors: E_2_ (10 nmol/L, Sigma-Aldrich), P_4_ (1 μmol/L, Sigma-Aldrich), recombinant human EGF (10 ng/mL, R&G, Minneapolis, MN), recombinant human bFGF (10 ng/mL, Genzyme TECHNE, Minneapolis, MN) or recombinant human VEGF (10 ng/mL, Sigma-Aldrich). SP cells were also seeded at a clonal density of 400 cells/cm^2^ in 35 mm dishes and cultured in EGM-2MV medium. EGM-2MV medium is a custom medium for EC culture, containing endothelial cell basal medium-2 and endothelial cell growth supplements EGM-2MV SingleQuots (human EGF, hydrocortisone, GA-1000, FBS, VEGF, human bFGF, insulin-like growth factor-I, and ascorbic acid, heparin, Gentamicin and Amphotericin). MP cells were concurrently seeded and cultured under the same conditions as the SP cells. All cultures were incubated for 14 days in a humidified CO_2_ incubator at 37 °C in 5% CO_2_, and the medium was changed every three to four days. After 14 days, clusters of cells were considered colonies when they were visible macroscopically and had more than 50 cells. Colonies were counted and the cloning efficiency (CE) was determined from the formula: CE (%) = (number of colonies/number of cells seeded) × 100.

***Co-culture of SP and MP cells***

To track the fate of SP cells in co-culture with MP cells, the SP and MP cells were labeled with two different fluorescent dyes - Vybrant DiO (green fluorochrome) and Vybrant DiI (red fluorochrome) (Molecular Probes, Eugene, OR). These fluorochromes are lipophilic carbocyanine dyes binding to intracellular phospholipid bilayer membranes. They are non-translocating dyes with low cytotoxicity which make them particularly suitable for long-term labeling and tracking of cells. Due to their high molecular weight they are considered to be non-gap junction permeable. 3,3'-dioctadecyloxacarbocyanine perchlorate (DiO) and 1,1'-dioctadecyl-3,3,3,'3,'-tetramethylinocarbocyanine perchlorate (DiI) have different maximal excitation and emission energies, 549/565 and 484/501 nm, respectively. These reagents allow two-color labeling of cell populations for identification after mixing and co-culture.

Cells were labeled according to the manufacturer's instructions. Briefly, SP and MP cells were suspended at a density of 1x10^6^/ mL in DMEM containing 1% antibiotic-antimycotic solution, and 5 μL of DiO and DiI cell-labeling solutions were added per mL of cell suspension. These suspensions were incubated for 15 min at 37^o^C in the dark, followed by two washes with PBS. DiI-labeled SP cells and DiO-labeled MP cells were mixed at a ratio of 1:1 and seeded in 96 well plates. Co-cultures were maintained in DMEM^+^ in a humidified incubator at 37^o^C with 5% CO_2_.

***RT-PCR***

Total RNA was extracted from cell cultures using the RNeasy Mini Kit (Qiagen, Hilden, Germany) according to the manufacturer’s instructions and subjected to RT-PCR. **First-strand cDNA** was synthesized from 100 ng of total cellular RNA with a StrataScript First Strand cDNA Synthesis Kit (**Stratagene**, La Jolla, CA) and was amplified with 0.625U EX *Taq* polymerase (**Takara** Bio. Inc., Ohtsu, Japan) using specific PCR primers (Supplemental Table 2) according to the manufacturer’s recommendations. The PCR products were separated by electrophoresis on 2% agarose gels and visualized by 5% ethidium bromide staining with UV light illumination. The band intensity was evaluated by densitometry using NIH Image J 1.38 software (National Institutes of Health, Bethesda, Maryland, USA). The mRNA level was normalized to the expression of the housekeeping gene *GAPDH* and expressed as a ratio of the target gene mRNA concentration to *GAPDH* mRNA concentration in the corresponding total RNA derived from each sample.

## **Xenotransplantation and hormonal treatment**

The same number of SP and MP cells (10^4^ to 10^5^ cells), freshly isolated from human endometria, were immediately transplanted under the kidney capsules of NOG mice as described previously [2].

## ***Histology and immunohistochemistry***

Nephrectomy was performed for morphological and immunohistochemical analysis 10 weeks after transplantation. The graft-bearing kidneys were embedded in Tissue-Tek OCT compound (Sakura Finetech, Torrance, CA) and serially sectioned at 6 μm. Histological analysis was performed by staining with H&E (Sigma-Aldrich) for evaluation of the endometrial glandular and stromal structures.

**Cryosection**s (6 μm thickness) were transferred to microscope slides, air dried for 30 min and washed in PBS three times for 5 min. Sections were fixed in 100% acetone for 10 min on ice and washed again. Some sections, culture dishes and plates were fixed in 4% paraformaldehyde for 20 min at room temperature and **permeabiliz**ed with 0.1% **Triton** X-100 in PBS for 5 min. After blocking with 10% BSA for 30 min, they were incubated with the pre-titrated primary antibodies (Supplemental Table 3) for 60 min at room temperature and washed. For indirect immunofluorescence staining, the first antibodies were visualized by incubation with secondary antibodies conjugated with Alexa Fluor 488 (green) and 568 (red) (Molecular Probes, Inc.) for 60 min. For direct immunofluorescence staining, Cy3-conjugated vimentin antibody (clone V9) (Sigma Chemical) and Cy3-conjugated αSMA antibody (clone 1A4) (Sigma Chemical) (Supplemental Table 3) were used. After washing with PBS, the slides were mounted with VECTASHIELD containing 4',6-diamidino-2-phenylindole (DAPI) (Vector Laboratories, Burlingame, CA) or after nuclear staining with 1 μg/mL Hoechst 33258 (Sigma Chemical) **for 5 min**, the slides were washed and mounted with VECTASHIELD(Vector Laboratories). We classified the histological feature of the reconstituted tissue and its adjacent transplantation site into four subtypes (i.e., glandular, vascular endothelial, migrating endothelial, and stromal), based on their dominant type (Fig. 2C). The reconstitution rate of ESP and EMP cells was determined by the following formula: Reconstitution rates (%) = (number of a corresponding subtype /number of transplanted kidneys [ESP, n=24; EMP, n=21]) × 100.

Images were collected using a fluorescence microscope (PROVIS AX-70, Olympus, Tokyo, Japan) or an inverted Leica DMIRE2 fluorescent microscope (Leica Microsystems Inc., Wetzlar, Germany) equipped with a CCD camera (VB-700; Keyence Corp., Osaka, Japan) and Leica TCS SP2 operating system (Leica Microsystems Inc.).

## ***Statistics***

In all cases, data were averaged and used in subsequent analyses. Results are expressed as means ± SEM. Comparisons among the SP rates for each of the four phases were made with a Tukey test using SPSS software, version 14 (SPSS Inc, Chicago, IL). All other statistical comparisons were done using the unpaired Student’s t test. P values less than 0.05 were considered statistically significant.

**References**

1. Noyes RW, Hertig AT, Rock J (1950) Dating the endometrial biopsy. Fertil Steril 1: 3-25.

2. Masuda H, Maruyama T, Hiratsu E, Yamane J, Iwanami A et al. (2007) Noninvasive and real-time assessment of reconstructed functional human endometrium in NOD/SCID/γ_c_^null^ immunodeficient mice. Proc Natl Acad Sci U S A 104: 1925-1930.

3. Goodell MA, Brose K, Paradis G, Conner AS, Mulligan RC (1996) Isolation and functional properties of murine hematopoietic stem cells that are replicating *in vivo*. J Exp Med 183: 1797-1806.

4. Matsuzaki Y, Kinjo K, Mulligan RC, Okano H (2004) Unexpectedly efficient homing capacity of purified murine hematopoietic stem cells. Immunity 20: 87-93.

5. Ito M, Hiramatsu H, Kobayashi K, Suzue K, Kawahata M et al. (2002) NOD/SCID/gamma(c)(null) mouse: an excellent recipient mouse model for engraftment of human cells. Blood 100: 3175-3182.

6. Quintana E, Shackleton M, Sabel MS, Fullen DR, Johnson TM et al. (2008) Efficient tumour formation by single human melanoma cells. Nature 456: 593-598.
